# Mutation analysis of BCR-ABL1 kinase domain in chronic myeloid leukemia patients with tyrosine kinase inhibitors resistance: a Malaysian cohort study

**DOI:** 10.1186/s13104-024-06772-1

**Published:** 2024-04-20

**Authors:** Zahidah Abu Seman, Fadly Ahid, Nor Rizan Kamaluddin, Ermi Neiza Mohd Sahid, Ezalia Esa, Siti Shahrum Muhamed Said, Norazlina Azman, Wan Khairull Dhalila Wan Mat, Julia Abdullah, Nurul Aqilah Ali, Mohd Khairul Nizam Mohd Khalid, Yuslina Mat Yusoff

**Affiliations:** 1 Hematology Unit, Cancer Research Centre, Institute for Medical Research, National Institutes of Health, Ministry of Health Malaysia, Shah Alam, Selangor 40170 Malaysia; 2 Department of Pathology, Hospital Tunku Azizah, Ministry of Health Malaysia, Kuala Lumpur, Kuala Lumpur, WP 50300 Malaysia; 3 Centre for Medical Laboratory Technology Studies, Faculty of Health Sciences, Universiti Teknologi MARA, Puncak Alam, Selangor 42300 Malaysia; 4 Inborn Error of Metabolism and Genetic Unit, Metabolic & Cardiovascular Research Centre, Institute for Medical Research, National Institutes of Health, Ministry of Health Malaysia, Nutrition, Shah Alam, Selangor 40170 Malaysia; 5 Stem Cell and Regenerative Medicine Research Initiative Group, Universiti Teknologi MARA, Shah Alam, Selangor 40450 Malaysia

**Keywords:** Chronic myeloid leukemia, *BCR:ABL1* KD mutation, TKI resistance, Next generation sequencing, Sanger sequencing

## Abstract

**Objective:**

Mutational analysis of *BCR::ABL1* kinase domain (KD) is a crucial component of clinical decision algorithms for chronic myeloid leukemia (CML) patients with failure or warning responses to tyrosine kinase inhibitor (TKI) therapy. This study aimed to detect *BCR::ABL1* KD mutations in CML patients with treatment resistance and assess the concordance between NGS (next generation sequencing) and Sanger sequencing (SS) in detecting these mutations.

**Results:**

In total, 12 different *BCR::ABL1* KD mutations were identified by SS in 22.6% (19/84) of patients who were resistant to TKI treatment. Interestingly, NGS analysis of the same patient group revealed an additional four different *BCR::ABL1* KD mutations in 27.4% (23/84) of patients. These mutations are M244V, A344V, E355A, and E459K with variant read frequency below 15%. No mutation was detected in 18 patients with optimal response to TKI therapy. Resistance to TKIs is associated with the acquisition of additional mutations in *BCR::ABL1* KD after treatment with TKIs. Additionally, the use of NGS is advised for accurately determining the mutation status of *BCR::ABL1* KD, particularly in cases where the allele frequency is low, and for identifying mutations across multiple exons simultaneously. Therefore, the utilization of NGS as a diagnostic platform for this test is very promising to guide therapeutic decision-making.

**Supplementary Information:**

The online version contains supplementary material available at 10.1186/s13104-024-06772-1.

## Introduction

Chronic myeloid leukemia (CML) is a clonal myeloproliferative neoplasm characterized by the presence of the Philadelphia (Ph) chromosome derived from a reciprocal translocation between chromosome 9 and 22 (t(9;22)(q34;q11) to produce *BCR::ABL1* oncogene [1, 2]. Imatinib mesylate (IM) was the first *BCR::ABL1* tyrosine kinase inhibitor (TKI) approved as first-line therapy for CML due to its superior result in terms of response rates, progression-free and overall survival compared with the previous treatment options [3]. Despite the remarkable achievement of TKI therapy, 20–30% of patients developed a primary or secondary resistance to treatment during the disease course [4]. The most frequently described mechanism associated with resistance is the occurrence of point mutations in the *BCR::ABL1* gene [5–9]. Therefore, after the emergence of resistance and factors such as low compliance and inadequate dosing are excluded, *BCR::ABL1* KD mutation testing is indicated as per the recommendations of both ELN (European LeukemiaNet) and NCCN (National Comprehensive Cancer Network) [10–13].

Sanger sequencing (SS) is the recommended method for detecting *BCR::ABL1* KD mutation because of its high efficiency and accuracy [14]. However, the analytic sensitivity of this method is limited, with the detection of 15–20% mutations in the wild-type background [13, 15]. Hence, there is a need to explore other methods such as NGS (next generation sequencing), which offer advantages in sensitivity, throughput and accuracy to determine *BCR::ABL1* KD mutations. This study aimed to detect *BCR::ABL1* KD mutations in CML patients with treatment resistance and assess the concordance between NGS and SS in detecting these mutations.

## Methods

### Participants and setting

This cross-sectional study was conducted on samples from CML patients with TKI resistance and those exhibiting optimal responses to TKI treatment. This study included samples from CML patients who received treatment with either imatinib or a second-generation TKI such as nilotinib or dasatinib. Samples from patients with Ph-positive (Ph+) acute lymphoblastic leukemia (ALL), patients with atypical CML and those on non-TKI therapy were excluded from this study.

The response to TKI therapy is defined according to European LeukemiaNet guidelines [11]. Patients with TKI resistance or treatment failure were those who failed to achieve a complete hematological response (CHR), complete cytogenetic response (CCR) and major molecular response (MMR) during treatment. Optimal responders were those who attained MMR *BCR::ABL1* ≤ 1% and ≤ 0.1% at 6 and 12 months of TKI therapy, respectively. CHR was characterized by the normalization of peripheral blood counts, and the disappearance of palpable splenomegaly. CCR indicated the absence of Ph + cells in the karyotype. MMR or MR3 was defined as a *BCR::ABL1* IS (International Scale) transcript level ≤ 0.1%. MR4 and MR4.5 were defined as *BCR::ABL1* IS ≤ 0.01% and ≤ 0.0032% respectively.

Samples from the TKI-resistant group were identified from those received by the Diagnostic Laboratory Haematology at Institute for Medical Research (IMR), National Institute of Health (NIH) from January 2017 to December 2020. All samples in this group had previously undergone Sanger sequencing at the Diagnostic Laboratory Haematology, which was the referral laboratory in the country for routine SS to detect *BCR::ABL1* KD mutations. TKI responders patients were selected from the Pathology Laboratory at Hospital Tunku Azizah from January 2019 to December 2020. The Pathology Laboratory commonly received samples and conducted the detection and quantification of *BCR::ABL1* transcripts at the time of diagnosis or during follow-up for CML patients.

### Sample size estimation

The sample size for the study was determined using the OpenEpi software [16]. Based on the prevalence of *BCR::ABL1* mutations in previous studies (22.7% [17], 22.4% [18] and 32.5% [19] in CML patients with imatinib resistance in Malaysia, a prevalence of 22.4% was considered. Accordingly, considering confidence interval at 95% and marginal error of 5%, a minimum number of 80 samples was required for this study.

### cDNA synthesis

The archived RNA was quantified and checked for quality using NanoDrop 2000 spectrophotometer (Thermo Fisher Scientific). RNA was previously extracted using the QIAamp RNA Blood Mini kit (Qiagen). Then, total cellular RNA (1 µg/µl) was reverse transcribed to cDNA using SuperScript IV First Strand Synthesis System (Thermo Fisher Scientific).

### Mutational analysis of *BCR::ABL1* KD by Sanger sequencing

Sanger sequencing was performed upon double-step PCR amplification of the *BCR::ABL1* KD. The first-round amplification was performed using a forward primer on exon 12/13 of the *BCR* gene and a reverse primer on exon 10 of the ABL1-R gene. A 0.5 µl of the first PCR product was used as a template in second amplification of PCR. The *ABL1* KD was amplified using three partially overlapping fragments, using set of primer that covers exon 4 until exon 10 *ABL1* gene [20].

Sanger sequencing was performed on ABI3730XL 96 capillary Genetic Analyzer using BigDye® Terminator v3.1 Cycle Sequencing kit. The obtained sequences were visualized and aligned with Genbank reference sequence NM_005157.5 using CLC Main Workbench, version 7.0.2 (Qiagen).

### Mutational analysis of *BCR::ABL1* KD by next-generation sequencing

Semi-nested PCR was performed to amplify the *BCR::ABL1* allele and followed by another PCR for ABL1 KD amplification using appropriate primers [20]. A total of 400ng of each purified PCR product was used as input for tagmentation and library preparation using the Illumina DNA Prep kit as per manufacturer instructions. The library was normalized to 2 nM and pooled for sequencing on an Illumina MiSeq. The FASTQ files generated were then uploaded to Illumina BaseSpace Sequence Hub (BSSH). These files were analyzed using DRAGEN RNA (Illumina Inc.) and produced an output VCard File (VCF) file for downstream analysis. The details of methodology for NGS are provided in the supplemental Methods.

## Results

This study includes 84 CML patients who either do not respond or have lost their response to TKI therapy, as well as 18 CML patients who have achieved stable optimal responses to TKI therapy. The characteristics of patients are listed in Table 1.

**Table 1:** Characteristics of patients

Characteristics	TKI resistant patients (*n* = 84)	Patients with optimal response (*n* = 18)
Median age, years	46 (15–72)	55 (19–65)
Gender, *n* (%)		
Male	46 (54.8)	11 (61.1)
Female	38 (45.2)	7 (38.9)
Race, *n* (%)		
Malay	49 (58.3)	13 (72.2)
Chinese	17 (20.2)	4 (22.2)
Indian	14 (16.7)	0 (0)
Others	4 (4.8)	1 (5.6)
CML phase, *n* (%)		
Chronic phase	56 (66.7)	18 (100)
Accelerated phase	18 (21.4)	0 (0)
Blast phase	10 (11.9)	0 (0)

### Detection of *BCR::ABL1* KD mutation

**Fig. 1:**
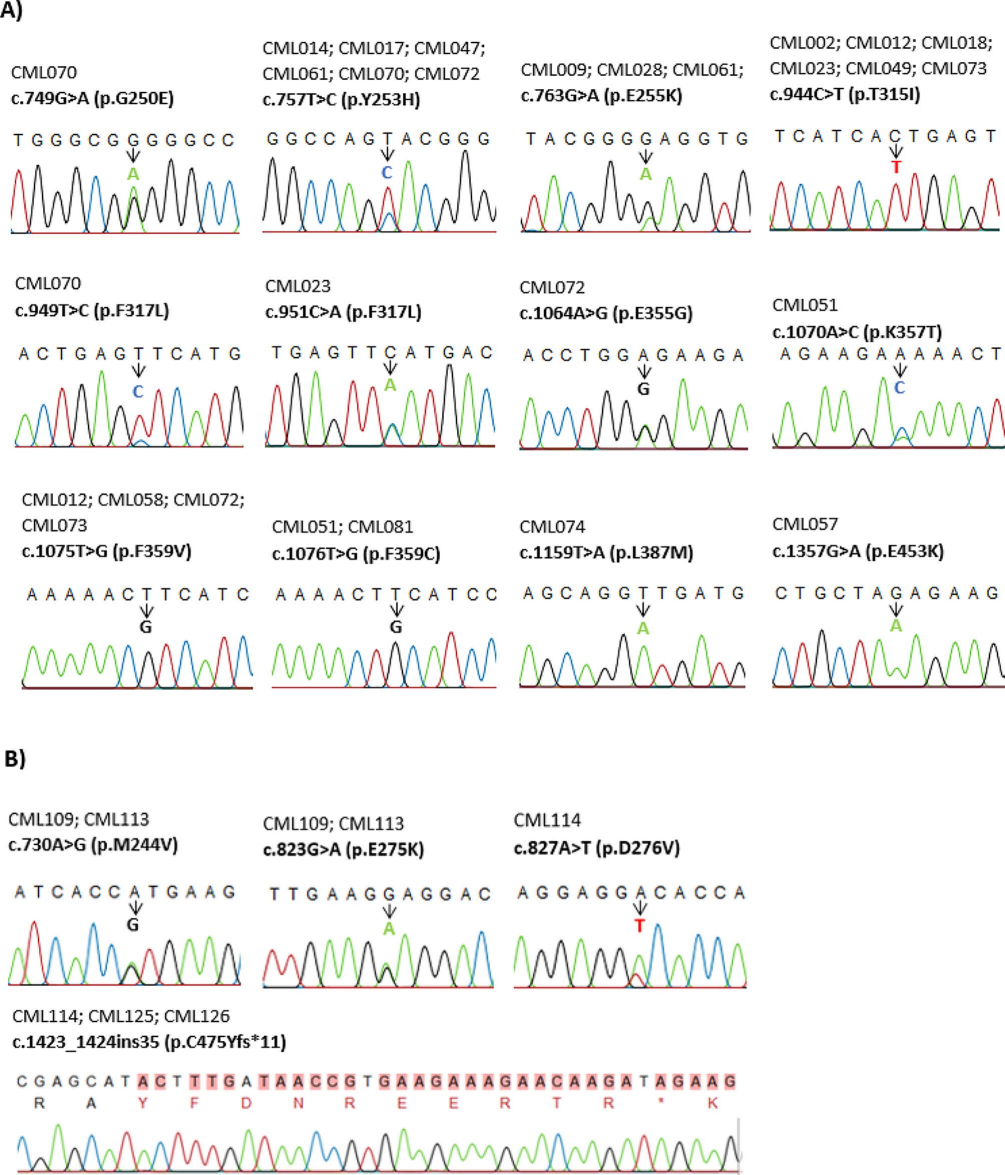
Sequencing chromatogram of variants detected in TKI resistant patients by Sanger sequencing. The ID number each of patient is provided at the beginning of each panel

**Fig. 2:**
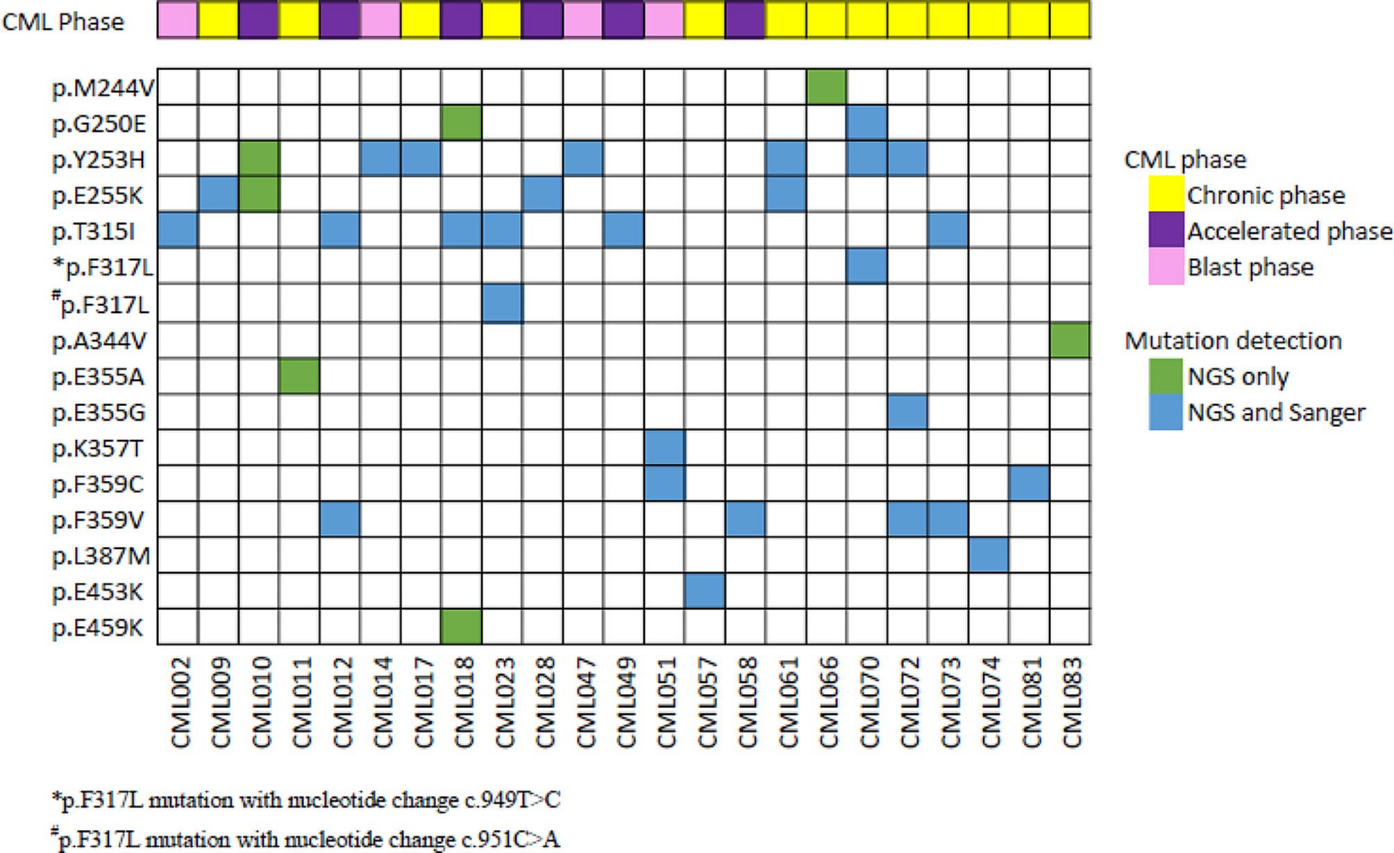
Distribution of variants detected by NGS and SS in TKI-resistant patients

In total, 12 different *BCR::ABL1* KD mutations were identified in 22.6% (19/84) of patients who were resistant to TKI treatment by SS (Fig. 1). NGS resulted in the detection of 16 different missense mutations in 27.4% (23/84) of TKI resistant patients (Fig. 2). Among the TKI-resistant patients, 14 carried one mutation, while 9 carried more than one mutation. NGS identified all true high-frequency mutations (> 15% frequency) found by SS and additional four low-frequency mutations (3 to 15% frequency). The additional mutations identified by NGS are M244V, A344V, E355A, and E459K (Table S1). No mutation was detected in patients with optimal response to TKI therapy by SS and NGS method.

### Variant classification

Variants p.M244V, p.G250E, p.Y253H, p.E255K, p.T315I, p.F317L, p.E355G, p.F359C, and p.F359V have been classified as pathogenic/likely pathogenic by ClinVar. These mutations have a phenotypic association with CML and lymphoblastic leukemia. Notably, variant p.F317L identified in this study has two different nucleotide changes, c.949T > C and c.951 C > A. The variant p.F317L with nucleotide change c.951 C > A was classified as likely pathogenic while variant c.949T > C p.F317L was classified as VUS by ClinVar. Notably, six mutations (p.L387M, p.E453K, p.E459K, p.A344V, p.E355A, and p.K357T) identified in this study were not documented in ClinVar.

**Fig. 3:**
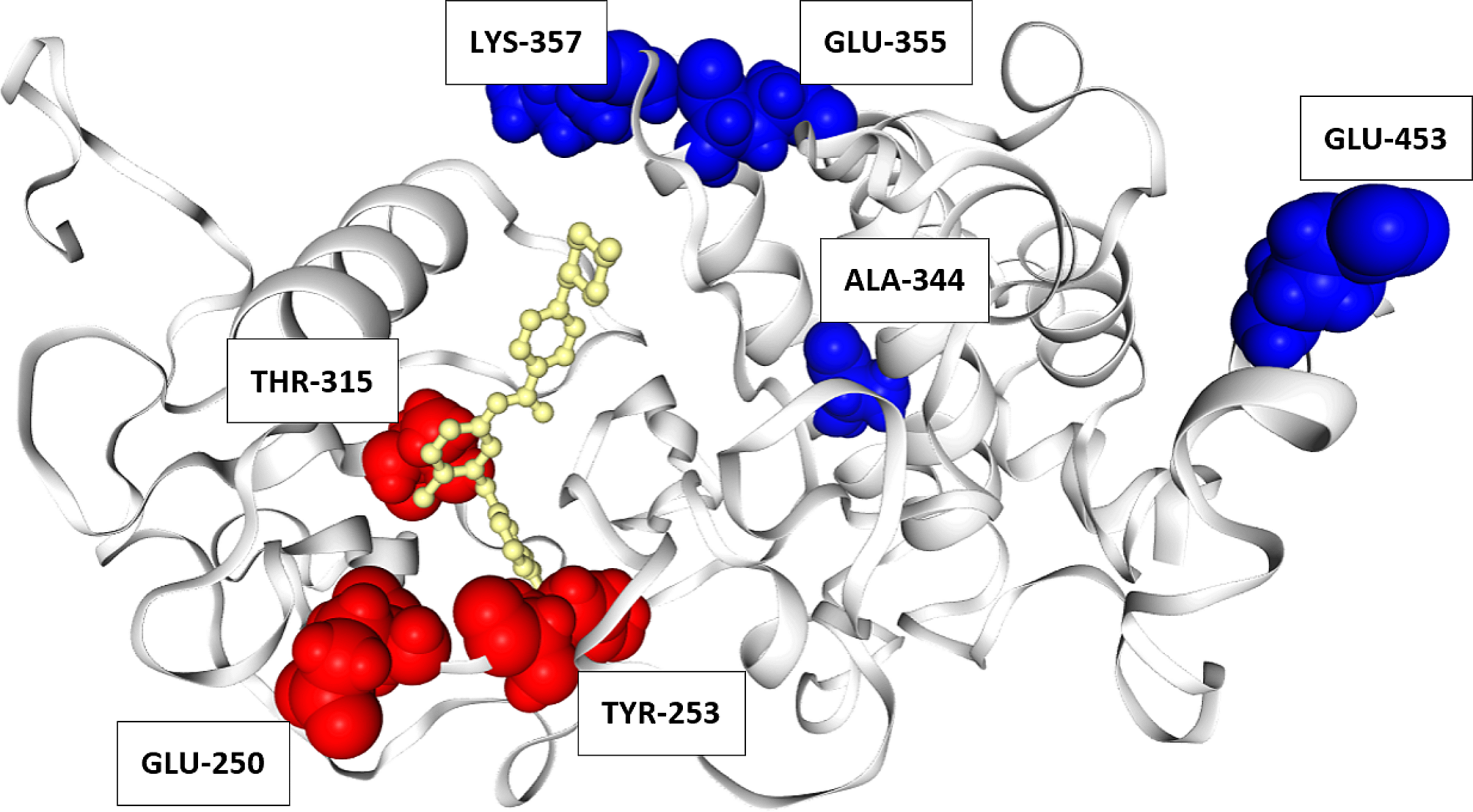
Location of missense mutations mapped onto the protein structure of ABL kinase domain. Blue indicates location of mutations A344V, E355A, K357T, and E453K; red indicates location of clinically resistant mutations; yellow indicates location of imatinib

In addition, Table 2 presents a list of mutations identified in current research and their corresponding sensitivity levels to the approved TKIs based on the previous study [12, 21–33]. The degree of sensitivity of mutations p.A344V, p.E355A, p.K357T, and p.E453K to TKI treatment has not been well established in clinical studies. Therefore, resistance profiles of these mutations were predicted using the web-based tool, SUSPECT-ABL (https://biosig.lab.uq.edu.au/suspect_abl/) [34]. Upon testing, all mutations were determined to be susceptible to imatinib, exhibiting change in protein stability, ddG (Delta Delta G) values of 0.19 for p.A344V, 0.28 for p.E355A, 0.19 for p.K357T and 0.37 for p.E453K. Figure 3 shows the locations of mutations and imatinib in ABL kinase domain.

**Table 2:** List of *BCR::ABL1* KD mutations identified in the present study that associated with TKIs resistance

TKI	Sensitive mutants	Resistant mutants
Imatinib		M244V G250E Y253H E255K T315I F317L E355G F359V/C E459K
Nilotinib	M244V F317L L387M	Y253H E255K T315I F359V/C
Dasatinib	M244V Y253H F359V/C L387M E459K	E255K T315I F317L
Bosutinib	M244V E255K Y253H F317L E355G	G250E T315I
Ponatinib	M244V T315I F317L	E255K

## Discussion

In this study, we described the development of laboratory assays using PCR and NGS-based approaches for *BCR::ABL1* KD mutation detection. The NGS amplicon sequencing involves generating libraries from PCR products, pooled and subsequently sequenced on the Illumina MiSeq platform.

NGS assay identified 16 different *BCR::ABL1* KD mutations in 27.4% (23/84) TKI-resistant patients. The variants detected at a frequency of 20% and above within *BCR::ABL1* transcripts were categorized as ‘high-level’. In contrast, variants detected at a frequency less than 20% but greater than or equal to 3% of *BCR::ABL1* transcripts were classified as ‘low-level’ [35]. In this study, Sanger sequencing was able to detect mutations with variant read frequency of more than 15%, therefore, identified 12 different mutations in 22.6% (19/84) TKI-resistant patients.

The current investigation shows the frequency of mutations by NGS detection in TKI-resistant patients is lower than in other studies, as reported 80% by Soverini et al. in 2016 [35], 33% by Duong et al. in 2017 [36], 59% by Erbilgin et al. in 2019 [37] and 30.9% by Liu et al. in 2020 [38]. According to a previous study by Kim et al. (2009), the proportion of patients in each phase of CML disease could affect the rate of mutations. The study found that a significant number of patients (63%) had *BCR::ABL1* KD mutations, which could be attributed to the high number of patients in the advanced phase (35% in the accelerated phase and 40% in the blast phase) [39]. Therefore, the large portion of patients in the chronic phase included in this study (66.7%) could explain the lower mutation rate compared with other studies. The research conducted on identifying *BCR::ABL1* KD mutation in Malaysian patients who are resistant to IM treatment was recorded at 22.4% [18], 22.7% [17], and 32.5% [19], which were consistent with our study. These previous studies have employed dHPLC followed by direct DNA sequencing as a mutation detection method.

The most common mutations type found in TKI-resistant patients by NGS detection were p.Y253H, p.T315I, p.E255K, and p.F359V. The occurrence of the mutations in the present study was reported as follows: p.Y253H in 8.3%, p.T315I in 7.1%, p.E255K in 4.8%, and p.F359V in 4.8%. The previous study conducted on the detection of *BCR::ABL1* KD mutations by NGS reported that p.Y253H mutation occurs in 8.2–14.4%, p.T315I in 4.1–23%, p.E255K in 4.1–10% and p.F359V in 5.6–6.6% which was comparable to the results obtained in the current study [35, 36, 38]. Among TKI-resistant patients in the present study, 9 individuals were found to have multiple mutations. Multiple mutations may reflect either polyclonal or compound mutations [40]. Previous studies have reported that multiple mutations were associated with worse outcomes and poor molecular response [29, 40, 41].

The majority of mutations identified in this study are situated distally from the imatinib-binding site (Fig. 3), making it challenging to explain their resistance mechanism. A new machine learning-based tool called SUSPECT-ABL is utilized to assess mutations with unknown effects on TKIs. These mutations were predicted to be susceptible to imatinib, which aligns with their location further away from the drug-binding site (Fig. 3). In contrast, clinically resistant mutations tend to cluster around imatinib (Fig. 3). Despite showing superior performance compared to other machine learning and molecular dynamics-based prediction [34], accuracy of SUSPECT-ABL is primarily high for residues in close proximity to imatinib. This highlights the limitations of current in silico tools and underscores the need to employ a better predictor for drug efficacy beyond measuring drug affinity alone.

In summary, the result of this study demonstrates that NGS is more sensitive than Sanger sequencing method for detecting *BCR::ABL1* KD mutations in CML patients, thus emphasizing the crucial role of NGS assays in advancing the understanding of mutation-associated TKI resistance in CML. The flexibility and capacity to assess multiple targets in a single run render NGS a valuable tool, considering its potential to revolutionize the detection and characterization of mutations in CML. Moreover, these findings indicate that integrating NGS into clinical practice has the potential to significantly impact therapeutic decision-making, ultimately leading to improved patient outcomes in the management of CML.

## Limitation

The current study could not prove that NGS able to detect emerging *BCR::ABL1* KD mutations earlier than Sanger sequencing. The present study also was not able to distinguish between polyclonal and compound mutations in *BCR::ABL1* KD since the long-range (LR)-NGS and fragment subcloning was not performed due to limited budget. The samples included in this study were selected from only a single center and thus the findings may not be representative of the general population of TKI resistant CML patients in Malaysia. Due to financial and time constraint, only 84 samples were able to recruit in the current study.

### Electronic supplementary material

Below is the link to the electronic supplementary material.

Supplementary Material 1

## Data Availability

The data are not publicly available due to ethical restrictions by the Medical Research Ethics Committee (MREC) Ministry of Health Malaysia (NMRR-19-3693-52311). A Table S1 has been attached. The remaining data are available from the corresponding author upon reasonable request, subject to the permission of Medical Research & Ethics Committee Ministry of Health Malaysia.

## Abbreviations

bp: base pairs
cDNA: complementary DNA
CML: Chronic myeloid leukemia
ddG: Delta Delta G
DNA: Deoxyribonucleic acid
ELN: European LeukemiaNet
IM: imatinib mesylate
IS: International Scale
KD: Kinase domain
NCCN: National Comprehensive Cancer Network
NGS: Next generation sequencing
PCR: Polymerase chain reaction
RNA: Ribonucleic acid
SS: Sanger sequencing
TKI: Tyrosine kinase inhibitor
VCF: VCard File
