# Supplementary material for: Mutation analysis of BCR-ABL1 kinase domain in chronic myeloid leukemia patients with tyrosine kinase inhibitors resistance: a Malaysian cohort study
Source: BMC Res Notes. 2024 Apr 20;17:111. doi: 10.1186/s13104-024-06772-1 (PMC11031984; doi:10.1186/s13104-024-06772-1)
Supplement: Supplementary file 1 — Supplementary Material 1 [file 13104_2024_6772_MOESM1_ESM.docx]

**Supplementary Methods**

**Library construction and Sequencing**

The first PCR for the sequenced region of *BCR::ABL1* was conducted as mentioned above. Then the *ABL1* KD was amplified at the second-round PCR from 0.5 µl of the previous PCR product, using forward primer on exon 4 *ABL1* gene and reverse primer on exon 10 *ABL1* gene (Soverini, 2007). The PCR product was purified using a QIAquick PCR purification kit (Qiagen) and measured concentration using the Qubit dsDNA HS Assay kit (Invitrogen).

A total of 400ng of each purified PCR product was used as input for tagmentation and library preparation using the Illumina DNA Prep kit as per manufacturer instructions. Tagmented DNA was purified and amplified with index adapters to identify each sample. Following bead-based clean-up steps, the library quality and size were checked with the Qubit dsDNA HS Assay kit (Invitrogen) and High Sensitivity DNA 1000 Kit (Agilent). The library was normalized to 2 nM and pooled for sequencing. The library pool was loaded at 10 pM with 1%PhiX (Illumina). Sequencing was carried out on an Illumina MiSeq Micro V2 flow cell in 150 bp read length in paired-end mode, reaching an average depth of coverage of 5000X.

#### Sequencing data analysis

The FASTQ files generated by MiSeq Reporter (Illumina) were quality-checked using Sequencing Analysis Viewer Software. The FASTQ files were then uploaded to Illumina BaseSpace Sequence Hub. These files were analyzed using DRAGEN RNA (Illumina Inc.) on the Illumina DRAGEN BioIT platform as per standard protocol and produced an output VCard File (VCF) file. The VCF files were used for downstream analysis on a BaseSpace Variant Interpreter, that provided genomics variant filtering and interpretation. The detection limit of mutation detection was validated at 3% to avoid false positive and false negative results.

The list of mutated genes was compared with the ClinVar (http://www.ncbi.nlm.nih.gov/clinvar) (Landrum et al., 2016), Catalog of Somatic Mutations in Cancer (COSMIC, http://cancer.sanger.ac.uk/cosmic) (Tate et al., 2018), and dbSNP (http://www.ncbi.nlm.nih.gov/projects/SNP/) (Sherry et al., 2001) to evaluate clinical significance of variants. The potential functional impacts of SNVs were assessed using the Sorting Intolerant from Tolerant (SIFT) (Sim et al., 2012) and Polymorphism Phenotyping v2 (PolyPhen-2) prediction algorithms (Adzhubei et al., 2010).

**Supplementary Results**

Table S1. Variants identified in TKI-resistant patients

| Sample ID | CML Phase | Nucleotide change  (NM_005157.6) | Amino acid change  (NP_005148.2) | Consequence | Clinical Significance (VarSome) | dbSNP | COSMIC IDs | NGS on MiSeq | | Genotype by  Sanger sequencing |
| --- | --- | --- | --- | --- | --- | --- | --- | --- | --- | --- |
|  |  |  |  |  |  |  |  | Variant Read Frequency | Total Read Depth |  |
| CML002 | Blast | c.944C>T | p.Thr315Ile (T315I) | Missense | Pathogenic / likely pathogenic | rs121913459 | COSM12560, | 0.766 | 2637 | Mutation detected |
| CML009 | Chronic | c.763G>A | p.Glu255Lys (E255K) | Missense | Likely pathogenic | rs121913448 | COSM12573 | 0.994 | 2910 | Mutation detected |
| CML010 | Accelerated | c.757T>C | p.Tyr253His (Y253H) | Missense | Pathogenic / likely pathogenic | rs121913461 | COSM12576 | 0.106 | 208 | No mutation detected |
|  |  | c.763G>A | p.Glu255Lys (E255K) | Missense | Likely pathogenic | rs121913448 | COSM12573 | 0.108 | 213 | No mutation detected |
| CML011 | Chronic | c.1064A>C | p.Glu355Ala (E355A) | Missense | NA | NA | NA | 0.030 | 2283 | No mutation detected |
| CML012 | Accelerated | c.944C>T | p.Thr315Ile (T315I) | Missense | Pathogenic / likely pathogenic | rs121913459 | COSM12560, | 0.994 | 2617 | Mutation detected |
|  |  | c.1075T>G | p.Phe359Val (F359V) | Missense | Likely pathogenic | rs121913452 | COSM12605 | 0.999 | 3061 | Mutation detected |
| CML014 | Blast | c.757T>C | p.Tyr253His (Y253H) | Missense | Pathogenic / likely pathogenic | rs121913461 | COSM12576 | 0.909 | 2727 | Mutation detected |
| CML017 | Chronic | c.757T>C | p.Tyr253His (Y253H) | Missense | Pathogenic / likely pathogenic | rs121913461 | COSM12576 | 0.593 | 2071 | Mutation detected |
| CML018 | Accelerated | c.749G>A | p.Gly250Glu (G250E) | Missense | Likely pathogenic | rs121913453 | COSM12577 | 0.086 | 1552 | No mutation detected |
|  |  | c.944C>T | p.Thr315Ile (T315I) | Missense | Pathogenic / likely pathogenic | rs121913459 | COSM12560, | 0.635 | 490 | Mutation detected |
|  |  | c.1375G>A | p.Glu459Lys (E459K) | Missense | NA | rs1064156 | COSM12634 | 0.050 | 1031 | No mutation detected |
| CML023 | Chronic | c.944C>T | p.Thr315Ile (T315I) | Missense | Pathogenic / likely pathogenic | rs121913459 | COSM12560, | 0.309 | 404 | Mutation detected |
|  |  | c.951C>A | p.Phe317Leu (F317L) | Missense | Likely pathogenic | rs121913451 | COSM131573 | 0.632 | 486 | Mutation detected |
| CML028 | Accelerated | c.763G>A | p.Glu255Lys (E255K) | Missense | Likely pathogenic | rs121913448 | COSM12573 | 0.510 | 2380 | Mutation detected |
| CML047 | Blast | c.757T>C | p.Tyr253His (Y253H) | Missense | Pathogenic / likely pathogenic | rs121913461 | COSM12576 | 0.997 | 2298 | Mutation detected |
| CML049 | Accelerated | c.944C>T | p.Thr315Ile (T315I) | Missense | Pathogenic / likely pathogenic | rs121913459 | COSM12560, | 0.740 | 2366 | Mutation detected |
| CML051 | Blast | c.1076T>G | p.Phe359Cys (F359C) | Missense | Likely pathogenic | rs1057519775 | COSM1732691 | 0.732 | 1189 | Mutation detected |
|  |  | c.1070A>C | p.Lys357Thr (K357T) | Missense | NA | NA | NA | 0.720 | 1266 | Mutation detected |
| CML057 | Chronic | c.1357G>A | p.Glu453Lys (E453K) | Missense | NA | NA | COSM3375127 | 0.833 | 874 | Mutation detected |
| CML058 | Accelerated | c.1075T>G | p.Phe359Val (F359V) | Missense | Likely pathogenic | rs121913452 | COSM12605 | 0.436 | 2174 | Mutation detected |
| CML061 | Chronic | c.757T>C | p.Tyr253His (Y253H) | Missense | Pathogenic / likely pathogenic | rs121913461 | COSM12576 | 0.149 | 1765 | Mutation detected |
|  |  | c.763G>A | p.Glu255Lys (E255K) | Missense | Likely pathogenic | rs121913448 | COSM12573 | 0.195 | 1822 | Mutation detected |
| CML066 | Chronic | c.730A>G | p.Met244Val (M244V) | Missense | Likely pathogenic | rs121913456 | COSM12608 | 0.064 | 2323 | No mutation detected |
| CML070 | Chronic | c.749G>A | p.Gly250Glu (G250E) | Missense | Likely pathogenic | rs121913453 | COSM12577 | 0.504 | 2337 | Mutation detected |
|  |  | c.757T>C | p.Tyr253His (Y253H) | Missense | Pathogenic / likely pathogenic | rs121913461 | COSM12576 | 0.258 | 2471 | Mutation detected |
|  |  | c.949T>C | p.Phe317Leu (F317L) | Missense | Other | rs1057519773 | COSM49074 | 0.187 | 3264 | Mutation detected |
| CML072 | Chronic | c.1064A>G | p.Glu355Gly (E355G) | Missense | Likely pathogenic | rs121913450 | COSM12611 | 0.659 | 2579 | Mutation detected |
|  |  | c.1075T>G | p.Phe359Val (F359V) | Missense | Likely pathogenic | rs121913452 | COSM12605 | 0.340 | 2636 | Mutation detected |
|  |  | c.757T>C | p.Tyr253His (Y253H) | Missense | Pathogenic / likely pathogenic | rs121913461 | COSM12576 | 0.999 | 2204 | Mutation detected |
| CML073 | Chronic | c.1075T>G | p.Phe359Val (F359V) | Missense | Likely pathogenic | rs121913452 | COSM12605 | 0.999 | 3650 | Mutation detected |
|  |  | c.944C>T | p.Thr315Ile (T315I) | Missense | Pathogenic / likely pathogenic | rs121913459 | COSM12560, | 0.999 | 3652 | Mutation detected |
| CML074 | Chronic | c.1159T>A | p.Leu387Met (L387M) | Missense | NA | NA | COSM131574 | 0.969 | 2237 | Mutation detected |
| CML081 | Chronic | c.1076T>G | p.Phe359Cys (F359C) | Missense | Likely pathogenic | rs1057519775 | COSM1732691 | 0.995 | 3276 | Mutation detected |
| CML083 | Chronic | c.1031C>T | p.Ala344Val (A344V) | Missense | NA | NA | NA | 0.053 | 2081 | No mutation detected |
